# The Terminal End Bud: the Little Engine that Could

**DOI:** 10.1007/s10911-017-9372-0

**Published:** 2017-02-06

**Authors:** Ingrid S. Paine, Michael T. Lewis

**Affiliations:** 1 Department of Molecular and Cellular Biology, Baylor College of Medicine, Houston, TX 77030 USA; 2 Department Radiology, Baylor College of Medicine, Houston, TX 77030 USA

**Keywords:** Terminal end bud, Mammary gland, Ductal elongation, Cap cell

## Abstract

The mammary gland is one of the most regenerative organs in the body, with the majority of development occurring postnatally and in the adult mammal. Formation of the ductal tree is orchestrated by a specialized structure called the terminal end bud (TEB). The TEB is responsible for the production of mature cell types leading to the elongation of the subtending duct. The TEB is also the regulatory control point for basement membrane deposition, branching, angiogenesis, and pattern formation. While the hormonal control of TEB growth is well characterized, the local regulatory factors are less well understood. Recent studies of pubertal outgrowth and ductal elongation have yielded surprising details in regards to ongoing processes in the TEB. Here we summarize the current understanding of TEB biology, discuss areas of future study, and discuss the use of the TEB as a model for the study of breast cancer.

## Overview of Mammary Gland Development

**Fig. 1:**
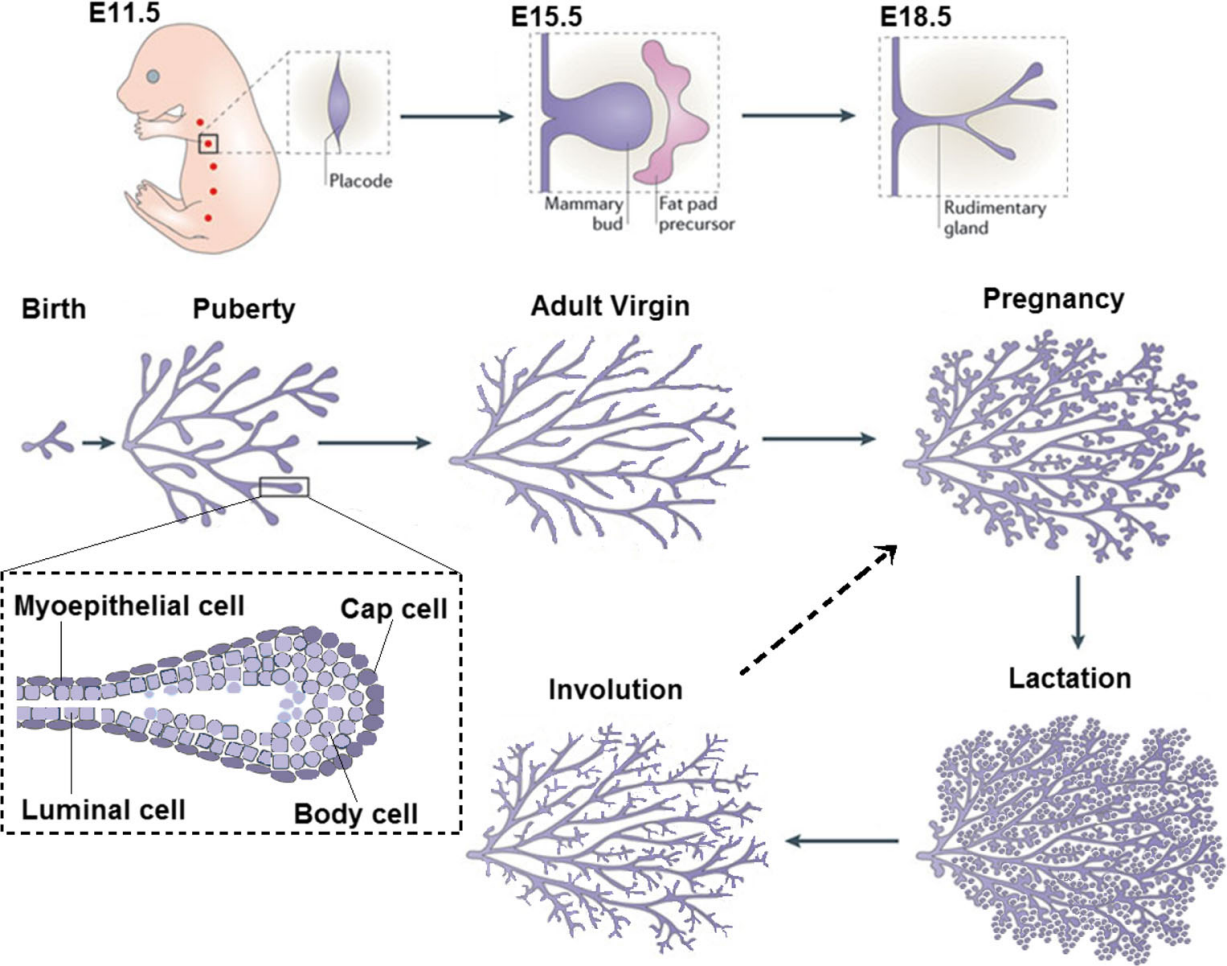
Overview of Mammary Development. Mammary placodes are formed on E11.5. The mammary bud sinks into the fat pad precursor on E15.5 and expands to form a rudimentary duct by E18.5. The gland remains relatively quiescent until the onset of puberty, when terminal end buds are formed and direct ductal elongation (blow up of pubertal time point). During pregnancy alveolar budding and differentiation take place to give rise to the milk producing cells. Adapted from [146]

In mammals, development of the mammary gland begins in the embryo with the formation of bilateral milk lines, or mammary ridges, running anterior to posterior displaced off of the ventral midline, beginning approximately on embryonic day 10.5 (E10.5) in the mouse [1]. At about E11.5 the lines transition into five pairs of lens shaped mammary placodes that mark the ultimate location of each nipple (Fig. 1). The placodes then evolve to form bulbs of epithelial cells that eventually invade into the underlying mesenchyme around E13.5. At this time, the underlying mesenchyme undergoes differentiation to form the condensed mammary mesenchyme through which the initial mammary bud must invade [2]. The mammary mesenchyme plays an important role in the regulation of sexual dimorphism, and is responsible for the androgen-mediated condensation of mesenchyme around the primary duct resulting in the elimination of the duct and the prevention of mammary epithelial growth in males [3].

The mammary buds themselves remain relatively growth quiescent until about E15.5, at which point hormone-independent cell proliferation drives bud elongation to form a mammary sprout. This sprout grows out of the condensed mammary mesenchyme and invades into the underlying fat pad precursor mesenchyme to form a solid chord of epithelial cells called the rudimentary mammary sprout. Subsequently, a lumen is formed in the sprout [4] and limited branching occurs, giving rise to a rudimentary ductal tree by about E18, which remains relatively quiescent until puberty at approximately 3–4 weeks of age (Fig. 1) [5, 6].

**Fig. 2:**
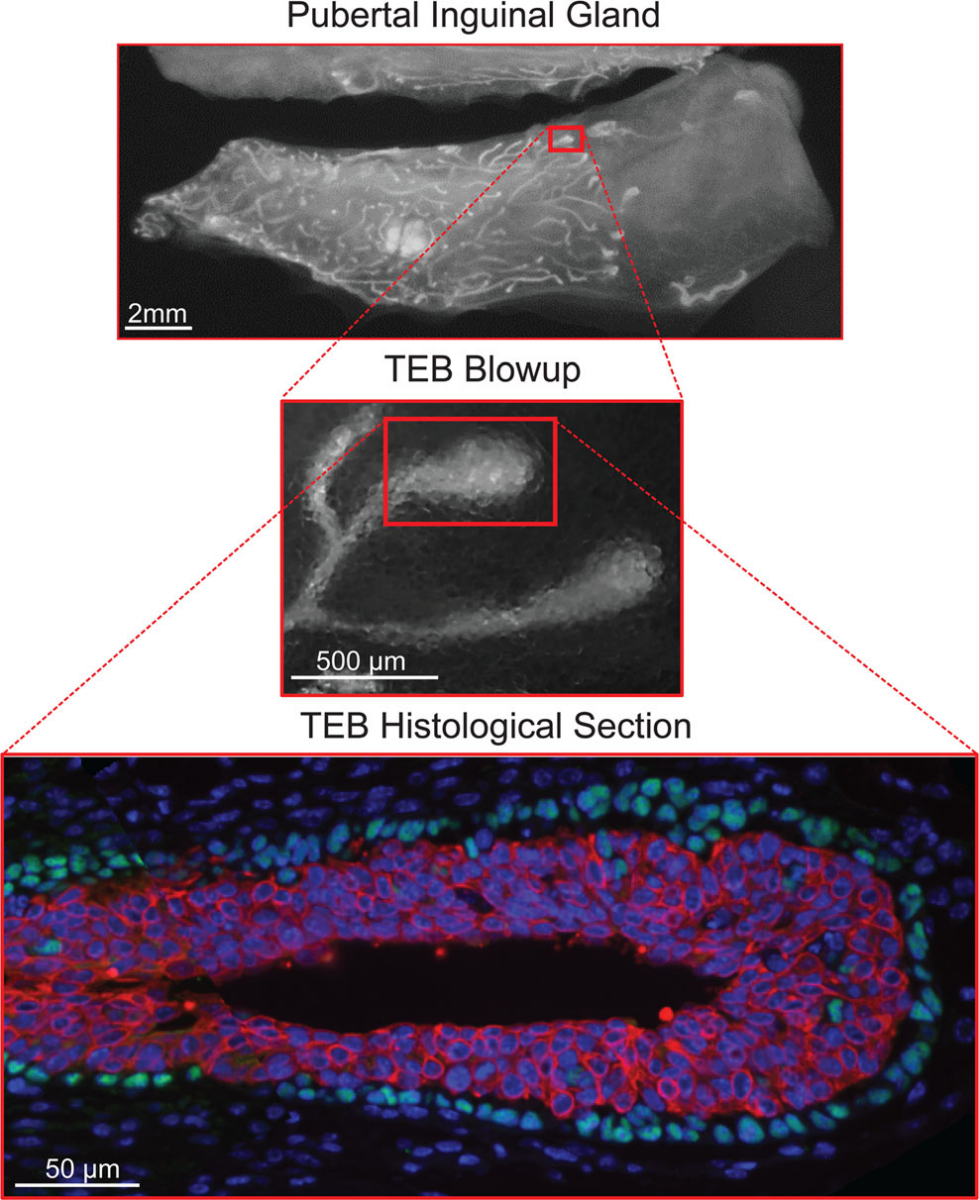
Terminal End Bud Whole Mounts and Histology. During puberty, terminal end buds direct the growth of the duct through the fat pad and are seen at the leading edge of the growing duct (*top panel*). The terminal end buds are bulb shaped structures that undergo regular bifurcation events (*middle panel*). A histological section of a terminal end bud reveals two compartments that can be distinguished by expression of cell surface markers (*bottom panel*)

At the onset of puberty, increased ovarian production of estrogen and pituitary gland production of growth hormone (among other systemic factors) promote cell division and the formation of terminal end buds (TEBs). TEBs are bulb-shaped structures unique to the mammary gland that direct the growth of the ducts throughout the rest of the fat pad (Fig. 2) [7, 8]. Regular bifurcation and branching during ductal elongation produces the main ductal system of the mammary tree [8]. Once the TEBs reach the edges of the fat pad they regress. Further side branching occurs off of the previously formed ducts during subsequent estrous cycles in response to progesterone signaling in the adult mammal but the growth of these side-branches is not driven by TEBs.

Additional development occurs upon pregnancy, when systemic progesterone and prolactin (among others) causes rapid proliferation of the mammary epithelium to form alveolar buds (Fig. 1) [9]. During pregnancy, the cells within alveoli undergo complete differentiation, polarize, and form the secretory alveoli capable of producing milk proteins and cytoplasmic lipid droplets. The precipitous reduction of progesterone in the context of continued presence of prolactin at parturition activates the secretory function of these cells characterized by movement of milk proteins and milk fat globules (membrane-bound cytoplasmic lipid droplets) into the lumen [10, 11].

Upon weaning, most epithelial cells in the differentiated alveoli undergo apoptosis and are cleared from the gland by both macrophages and a subset of surviving mammary epithelial cells [12, 13]. The gland undergoes progressive remodeling back to a virgin-like state through a process collectively called “involution” (Fig. 1). The process of alveolar differentiation, lactation, and involution can occur repeatedly while the animal is capable of reproducing, making the mammary gland one of the most regenerative organs in the body.

## Overview of Terminal End Buds across Species

Terminal end buds have been described in rodents (e.g. mice, rats), as well as in humans and other primates, but not in ruminants [7, 8, 14–17]. Ruminants instead have terminal ductal units (TDU) which direct the elongation and branching of the ducts during puberty and resemble a multi-lobular TEB, with each ductule growing from a central chord of epithelial cells typically 4–5 cells thick [18]. Due to the general lack of availability of human pubertal tissue the majority of work on TEBs has been done in rodents. However, a number of tissue samples from teenage women indicate TEB structures that are similar to those of rodents [19, 20]. Across species, TEBs are described as bulbous, highly proliferative, hormone dependent, structures that appear on the ends of growing ducts. The focus of this review describes the knowledge gained from the collective work done in mice.

## Terminal End Bud Biology

The terminal end bud (TEB) is a structure unique to the peri-pubertal mammary gland [8]. At the onset of puberty, the TEBs emerge from the stratified epithelium of the immature gland. Multiple vertical apical divisions of the luminal layer are responsible for the stratification of the simple bilayer into a bulbous TEB structure [21]. The TEBs invade through the fat pad and undergo regular bifurcation events to give rise to the primary mammary ductal tree (Fig. 2). Once the TEB reaches the edges of the fat pad, the TEBs regress completely [22], leaving behind blunt-ended ductal termini or sometimes smaller rounded buds which are frequently mistaken for TEBs but do not exhibit the histological structure or high levels of proliferation indicative of active TEBs. The TEBs are of special interest due to their unique characteristics including a heterogeneous cellular composition, high proliferation (60–90%) and apoptosis rates (5–15%), invasive ability, angiogenic properties, and their ability to recruit stromal cells [23].

### Structure

**Fig. 3:**
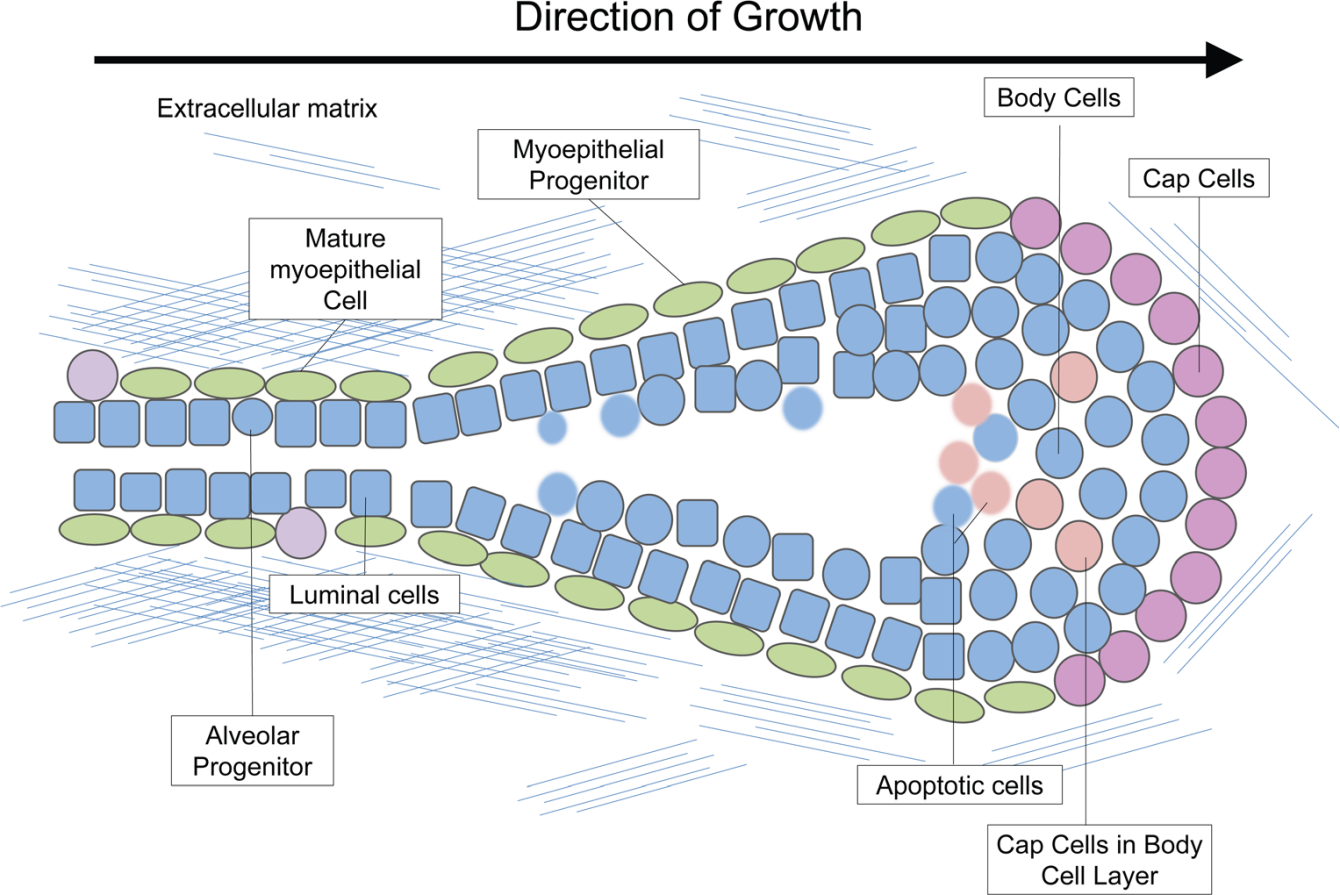
Terminal End Bud. The terminal end bud is a bulbous structure made up of two main compartments known as the cap and body cell layers. The cells in the front of the terminal end bud (pink) are least differentiated (cap cells and body cells), whereas cells become more differentiated in the narrowing region (myoepithelial progenitors-green) and the neck of the terminal end bud (luminal cells- blue, mature myoepithelial cells-green). Some cap cells can be found in the body cell layer (beige). The extra-cellular matrix is light around the leading tip of the terminal end bud and becomes a complex meshwork in the neck of the terminal end bud. Apoptosis in the body cell layer contributes to lumen formation (faded blue and beige)

In the mouse, as in other mammals, the TEB is shaped generally like a light bulb, with the least differentiated and most proliferative cells located in the bulbous region, and the more differentiated, less proliferative cells in the neck and subtending duct (Fig. 2). The TEB is made up of two main compartments. The outer compartment is composed of a single-cell layer of “cap cells” at the growing tip of the TEB, which differentiate into myoepithelial cells as the duct elongates (Figs. 2 and 3). In the mouse, the inner compartment is a multi-cellular layer approximately 4–6 cells in thickness known as the “body cell” layer [8]. The body cell layer is thought to be made up primarily of luminal and alveolar progenitors whose progeny differentiate into more mature luminal cells as the duct elongates (Fig. 3).

The cells within the TEB are held together loosely with two types of cadherin-mediated adherens junctions. The body cells express E-cadherin, whereas the cap cell layer expresses P-cadherin [24], allowing these two compartments to act in a coordinated fashion but also independently. E-cadherin appears to be required for participation in ductal elongation, as cells lacking E-cadherin expression are excluded from developing glands [25, 26]. P-cadherin, counterintuitively, is required to maintain the organization of the body cell layer, as treatment of TEBs with function-blocking antibodies against P-cadherin cause body cells to disaggregate and fill the luminal space [24]. The interior body cells also contain desmosomes, which may function in the process of collective migration during ductal elongation [27, 28]. In the mature duct, the adhesion between luminal cells becomes much stronger with the addition of tight junctions and gap junctions, and myoepithelial cells adhere to the basement membrane through hemidesmosomes [25].

The light bulb shape of the TEB is due, in part, to constraints provided by the extracellular matrix (ECM). The TEB has a distinct ECM composition from the subtending and mature duct. While the bulbous tip of the TEB has a very thin basement membrane (~104 nm) made up primarily of laminin and collagen type IV, the basement membrane in the neck region of the TEB becomes a thicker (~1.4um), more defined meshwork that includes fibrillar collagen type IV, heparin sulfate proteoglycans, laminins 1 and 5 as well as fibronectin and vitronectin [29–32]. The basal layer of the TEB and mature duct is in direct contact with these ECM components.

### Cap Cells

Cap cells are located around the leading tip of the TEB (Fig. 3) and are thought to be a reservoir for regenerative mammary stem cells as they have a higher ability to form an entire ductal tree when transplanted as a purified population [33]. Cap cells primarily give rise to the myoepithelial cells enveloping the mature duct, but have also been hypothesized to contribute to the luminal lineage as they have been observed migrating into the body cell layer [8, 34]. Cap cells express several markers that can be used to identify them, along with their mature counterpart, myoepithelial cells. Some markers of the basal lineage include keratin 5 and 14, smooth muscle actin, and p63 (Fig. 2). Additionally, expression of a stem cell specific isoform of SH2-containing inositol 5′-phosphatase (sSHIP) appears to mark cap cells specifically, although the specific function of this isoform in the mammary gland is unknown [33].

### Body Cells

Body cells form the inner mass of the TEB and are thought to include ductal and alveolar progenitors (Fig. 3). The inner most cells are incompletely polarized while body cells adjacent to the basal layer are polarized and form cadherin-based adherens junctions, cells in the interior are loosely held together with desmosomes [28]. The body cell layer gives rise to the luminal cells lining the interior of the duct, and a subset are responsible for differentiating into alveoli during pregnancy and lactation [35, 36]. These cells express keratin 6, 8 (Fig. 2), and 18, and, although they are not competent to respond to ovarian hormones to form alveolar structures, a fraction of them do express the estrogen and progesterone receptors [37, 38]. The body cell layer has a high apoptotic index, which has historically been understood as the major mechanism of lumen formation [39]. However, we recently discovered that the majority of the apoptotic cells are of cap cell origin [40]. Thus, it remains unclear what effect this pattern of apoptosis has on lumen formation.

### Caps Cells as Potential Mammary Stem Cells

Due to the impressive regenerative capacity of the mammary gland, the presence of a resident stem cell population has long been acknowledged [41]. DeOme et al. functionally demonstrated the existence of this cell type with transplantation assays, in which they demonstrated that epithelial segments taken from anywhere in the mature gland were capable of recapitulating the entire functional gland when transplanted into a cleared fat pad [42]. Additionally, in a series of studies using limiting dilution transplantation of retrovirally-tagged mammary epithelial cells into virgin and pregnant females, it was demonstrated that a single cell was capable of re-growing the entire mammary gland, and that lineage restricted progenitors for alveolar outgrowths and ductal branching were also present in the mature duct [43–45]. It has been postulated that cap cells are examples of multipotent mammary stem cells based on observations made during time lapse videos and several studies acknowledging the presence of cells of basal lineage within the body cell layer [8, 46–48]. More recently, cap cells have been shown by limiting dilution transplantation to produce a full mammary gland more frequently than other basal lineage cells when transplanted [33]. However, transplantation of single cells may not faithfully recapitulate the function of cells in the intact gland and recent lineage tracing in vivo has led to contrary conclusions regarding the stemness of cap cells [34, 49]. Van Keumeulen et al. [49] first published lineage tracing data using K14, K8, and K18 promoters to label different cell populations during mammary development. They determined that an embryonic K14-Cre/Rosa-YFP labeled stem cell gives rise to all mammary epithelium, while in puberty these K14-rtTA/TetO-Cre/Rosa-YFP labeled cells mark a lineage restricted progenitor cell that gives rise only to myoepithelium. A second lineage tracing study by Rios et al. [34] used a confetti reporter under a bovine K5 promoter and showed that induction during puberty resulted in labeling of both cap and body cells in the terminal end bud. This induction also resulted in clonal populations containing both lineages after an 8 week chase, suggesting the existence of a population of bipotent cells in the TEB during ductal elongation.

Interestingly, the cap cell population also exhibits high levels of Wnt signaling, which is regarded as a hallmark of stemness in many tissues [50] and expression of Par3L, a regulator of polarity essential for maintenance of stemness in the mammary gland [51]. In contrast to these data, recent studies investigating the fate of cap cells that have migrated into the body cell layer indicates that these cap cells are undergoing apoptosis and are not contributing to both basal and luminal lineages [40]. Together these data concerning a bi-potent stem cell in the TEB during ductal elongation remain contradictory and warrant further specific study.

## Mammary Stroma and TEB Function

**Fig. 4:**
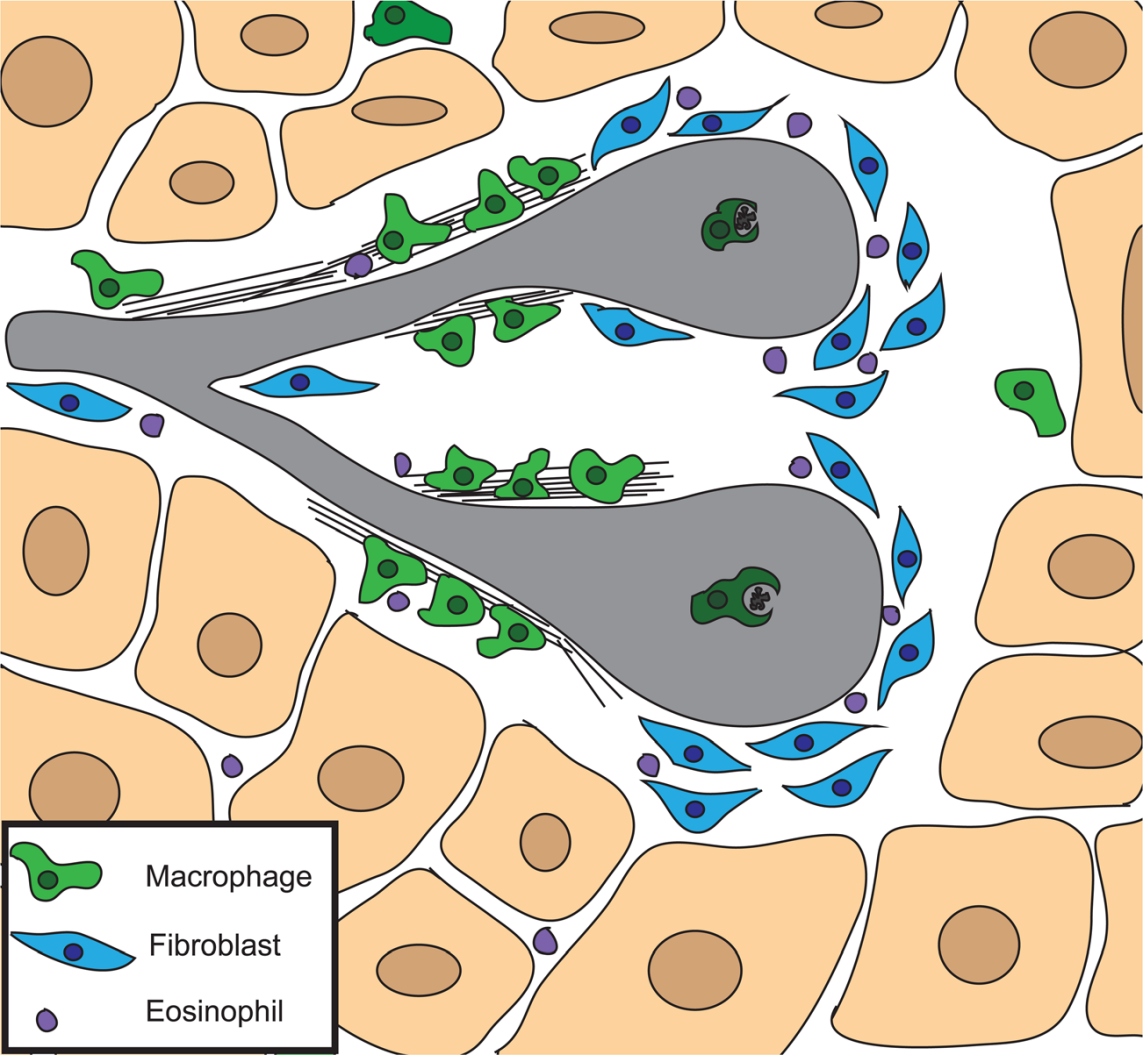
Stromal Cell Types Involved in Ductal Elongation. The mammary stroma contains many cell types that together are instructive for ductal morphogenesis. Three cell types are localized around active terminal end buds and are important for ductal elongation. Fibroblasts (*blue*) are found around the advancing tip of the terminal end bud and produce extracellular matrix proteins and growth factors. Macrophages (*green*) are found at the neck of the terminal end bud where they organize extracellular matrix proteins into a complex meshwork. Macrophages are also found within the body cell layer where they help to remove apoptotic bodies. Eosinophils (*purple*) are recruited to terminal end buds by eotaxin and are important for branching. The stroma also contains adipocytes (beige)

The mammary stroma, as a whole, is important for proper ductal morphology, and has been shown to be instructive for ductal morphogenesis. For example, mammary epithelium, transplanted into salivary mesenchyme, takes on the morphology of the salivary gland, although the cells maintain a mammary cell identity, as demonstrated by the production of milk proteins upon stimulation with pregnancy hormones [52]. The stroma from mice at different stages of the reproductive cycle also promote different aspects of duct development: stroma from virgin animals promotes branching and elongation, stroma from pregnant mice promotes alveolar differentiation, and stroma from involuting glands promotes epithelial apoptosis [52]. Therefore, the mammary stroma itself plays a role in ductal elongation and branching morphogenesis. Additionally, the mammary stroma is made up of several cell types including adipocytes, fibroblasts, macrophages, eosinophils, neutrophils, and endothelial cells. The TEB uniquely comes into direct contact with these cells in the stroma at its leading tip. Several stromal cell types, including fibroblasts, macrophages and eosinophils, are all localized around the TEB during ductal elongation (Fig. 4) [53–56].

### Fibroblasts

Fibroblasts are localized around the TEB and are responsible for the production of local growth factors, including fibroblast growth factors (FGFs), insulin-like growth factor-1 (IGF-1) and hepatocyte growth factor (HGF) when activated by systemic estrogen and growth hormones [56–58]. Growth factor signaling to the epithelium promotes the expansion of the luminal compartment during ductal elongation [53]. Fibroblasts also facilitate ECM production and degradation by producing laminin, collagen, fibronectin, proteoglycans, matrix metalloproteinases (MMPs), and tissue inhibitors of metalloproteinases (TIMPs) (Fig. 4) [56].

### Macrophages

Macrophages are localized to the TEB during ductal elongation. The macrophages are found around the neck of the TEB where they are recruited by the production of colony stimulating factor 1 (CSF1) by epithelial cells. Macrophages are also found within the body cell layer, where they contribute to lumen formation by clearing apoptotic cells via phagocytosis (Fig. 4) [55]. Depletion of the macrophage population results in decreased ductal outgrowth, branching, and TEB number, as well as changes in TEB morphology to a more rounded and blunt shape [54]. In the stroma, the macrophages localize mostly to collagen fibers and are thought to play an important role not only in the production of collagen I, but in its remodeling into long fibrillar bundles that project from the sides of the TEB in the direction of forward growth (Fig. 4) [54]. Macrophages are also involved in EGF signaling in the developing mammary gland which is discussed further below.

### Eosinophils

Eosinophils are found specifically around the leading tip of the TEB where they are recruited by the secretion of eotaxin by the TEB (Fig. 4) [55, 56]. Recently it has also been demonstrated that progesterone and estrogen induced amphiregulin (Areg) expression is sufficient to recruit eosinophils in ovariectomized mice [59]. Failure to recruit eosinophils to the TEB results in a decrease in branch number and TEB formation [55]. Interestingly, ductal elongation is not defective without eosinophils, indicating that the eosinophils may be more important for branch patterning than ductal elongation itself [55, 56]. Eosinophils are known to produce cytokines and growth factors including VEGF, although their exact function in the mammary gland is unknown [60].

## Signaling and TEB Function

Systemic signals required for proper morphology at each stage of mammary gland development has been well studied [61]. However, signaling within the TEB specifically, is still an area of ongoing investigation. While some factors are required for mammary gland development in general or at other stages, a few factors such as estrogen, FGF10, and GH are required for TEB formation, and other signaling molecules such as Wnt, SLIT, and TGFβ have subtle and sometimes divergent roles in TEB morphology and function.

### Estrogen

Estrogen, primarily from the ovary, can be secreted into the blood stream to act systemically on distant organs. Estrogen can also be synthesized locally in the mammary gland by adipose tissue and the enzyme aromatase [62]. In classical nuclear signaling, estrogen can bind its main receptor, estrogen receptor alpha (ESR1), causing dimerization and translocation into the nucleus, where it affects gene transcription. There is growing evidence for non-nuclear actions of ER at the plasma membrane as well [63, 64].

**Fig. 5:**
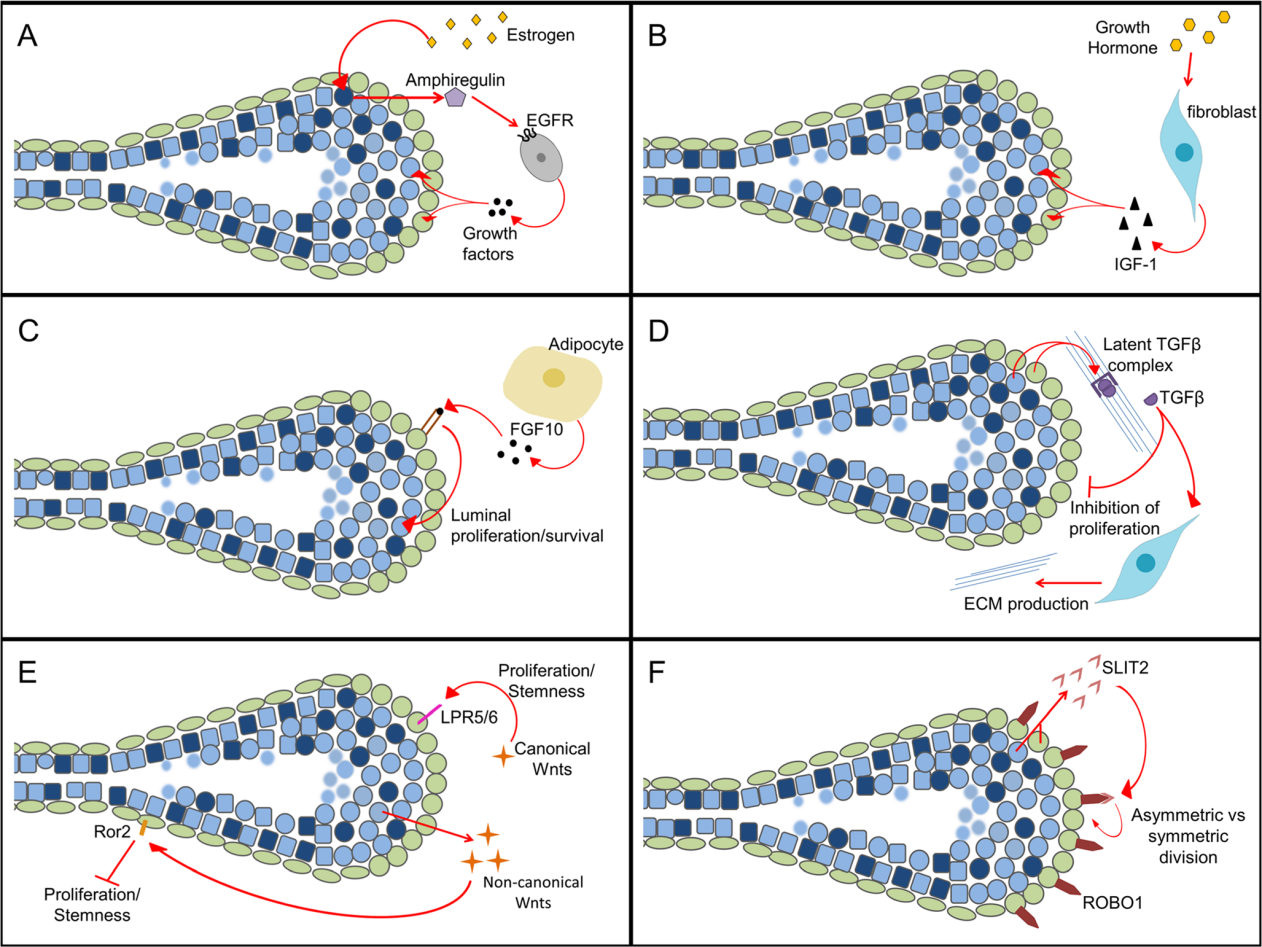
Signaling Essential for Terminal End Buds. **a** Estrogen from the ovaries signals through the Estrogen Receptor positive luminal cells to produce the paracrine factor amphiregulin, which acts on stromal cells to produce additional growth factors. **b** Growth hormone from the pituitary acts on fibroblasts near the terminal end bud to produce insulin growth factor which promotes proliferation in the epithelial cells. **c** The adipocytes produce fibroblast growth factor 10 which acts specifically on basal cells to induce a pro-proliferative response in the luminal compartment. **d** Transforming Growth Factor Beta is produced by the epithelial cells and secreted as a latent complex. Once cleaved it has divergent effects on stromal and epithelial cell types. **e** Canonical and non-canonical Wnt signaling oppose each other to control stemness in the mammary gland during puberty. **f** SLIT2 is produced by both luminal and basal cells in the terminal end bud and binds to the ROBO receptor found only in the basal compartment to promote survival of cap cells

Estrogen activity is important for sustained proliferation during ductal elongation and acts directly on the epithelium via ERα [65, 66]. Estrogen binds its receptor in a subset of luminal cells, which are then responsible for the production of amphiregulin (Areg). Areg is expressed as a transmembrane precursor that requires cleavage by ADAM17 in order to signal to nearby stromal cells. Areg signals in a paracrine manner to the stroma where it binds its receptor, epidermal growth factor receptor (EGFR), and causes additional expression of growth factors, including fibroblast growth factor (FGF) (Fig. 5a) [67]. Ectopic expression of Areg can rescue outgrowth in ERKO mice [68, 69]. Estrogen signaling alone can induce formation of some TEBs but estrogen in conjunction with growth hormone has a synergistic effect to form many TEBs [57]. While there are also activities of estrogen in the mammary stroma, as demonstrated by tissue recombination experiments and disruption of the ESR1 gene, these are not essential for ductal growth [70] .

### Progesterone

Progesterone (P_4_) is produced in the ovary by the granulosa and thecal cells in the corpus luteum. Once secreted, P_4_ can bind its receptor (PR) and regulate transcription of PR target genes [71], namely receptor activator of nuclear factor kappa B ligand (RANKL) which acts in a paracrine manner to induce proliferation and secretory fate in nearby PR- cells [72]. The progesterone receptor has two isioforms, PRA and PRB, although only PRB is required in the mammary gland [61].

The role of progesterone signaling in pubertal outgrowth is controversial. Several early studies indicated that PR is dispensible for ductal elongation but required for side branching and alveolar budding in the adult animal [73–75]. More recent work with IGF-1 KO mice treated with IGF-1 and estrogen or progesterone has demonstrated that PR signaling has a synergistic effect on ductal outgrowth, similar to that of estrogen [76]. In addition, reciprocal transplantation experiments indicate divergent roles for PR in the epithelium vs the stroma, with stromal PR being important for ductal outgrowth but not alveolar budding and epithelial PR being disspensible for ductal outgrowth but required for alveolar budding [77, 78]. Most recently, Aupperlee et al. [79] demonstrated that progesterone, although not required for ductal elongation, is sufficient to cause TEB formation and ductal outgrowth in ovariectomized mice. Authors claim is this probably due to overlapping regulation of Areg by both PR and ER [79].

### Growth Hormone

Growth Hormone (GH) is important at the onset of puberty to induce formation of TEBs. GH is produced by the pituitary and released into the blood stream in response to signaling from the hypothalamus, but GH can also be produced in the ovary [80].

GH promotes the formation of TEBs by acting primarily in the stroma via paracrine IGF-I signaling. The downstream effector of GH signaling, IGF-I, is required for TEB formation, and treatment of mice with GH or IGF-I alone will form a limited number of TEBs (Fig. 5b). However, the addition of estrogen signaling causes bursts of proliferation that is required for rapid ductal production during puberty [65, 70, 81–83]. After the formation of TEBs, IGF-1 is expressed in the stroma and within the TEB and is important for proper branching pattern but is not necessary for maintenance of the TEB itself [84].

### Epidermal Growth Factor Family

The epidermal growth factor receptor (EGFR) is a receptor tyrosine kinase (RTK). Binding of EGF to its receptor results in receptor dimerization and activation of the intracellular kinase domain with subsequent trans-auto phosphorylation. Downstream signaling includes wide ranging effects. EGFR activation of Ras/MEK leads to mitogenic signals; activation of PLC-**γ**1 regulates migration; and activation of PI3K-AKT leads to cell survival [85]. EGF family ligands are expressed as transmembrane precursors that require cleavage for receptor binding and activation. In the mammary gland ADAM and MMPs are responsible for EGF ligand cleavage [86, 87].

Both EGF and EGFR are expressed in the mammary gland during puberty, alveolar differentiation, and lactation [88]. During puberty, EGFR is highly expressed in the TEBs and the surrounding stroma [89]. Implantation of an EGF-containing pellet into growth quiescent glands of ovariectomized mice resulted in TEB formation and local outgrowth, however implantation of pellets into mice with intact ovaries did not result in increased growth [89]. Reciprocal transplantation studies of epithelium and stroma expressing a kinase-impaired form of EGFR (*waved-2*) determined that EGF signaling in the stroma is important for successful ductal outgrowth during puberty [90]. Further, expression of a dominant negative EGFR also caused decreased outgrowth and glands failed to form TEBs all together. However, upon pregnancy these animals successfully underwent alveologenesis and lactation suggesting a pregnancy associated rescue of the ductal outgrowth [91].

ErbB2 is highly expressed in the mammary epithelium during puberty. This receptor is best known for is proto-oncogene role in breast cancer, however, it also plays an important role in normal mammary gland development. ErbB3 is not capable of kinase activity and therefore requires hetero-dimerization with another receptor for downstream signaling. ErbB2 is thought to be its main partner in signaling due to their co-expression in breast cancers and because KO of Erbb4 has no effect on outgrowth during puberty [92]. Both ErbB2 and ErbB3, on the other hand, seem to be important for ductal elongation and TEB regulation. Both ErbB2 and ErbB3 KO mice die in utero, however when mammary buds were transplanted into WT animals Jackson-Fisher et al. found that the two receptors share a similar phenotype [93]. ErbB2 −/− and ErbB3−/− epithelium were both unable to fully fill the fat pad compared to the contralateral WT controls. Interestingly, ErbB3-null glands contained more TEBs (and more branchpoints), however they were of smaller size than WT controls, and regress by 20 weeks even without filling of the fat pad. Further, the TEBs of both KOs exhibit aberrant organization including multilayered cap cell regions, increased number of cap cells in the body cell layer, large gaps between the cap and body cell layers, and luminal filling [93]. Together these data are strong support for the cooperative signaling of ErbB3 and ErbB2 in the TEB during ductal elongation.

### Fibroblast Growth Factor

There are 23 FGF ligand family members but only a subset have been investigated in the mammary gland with FGFR signaling being required for normal development ([94, 95] and references therein). Canonical FGF subfamily members signal in a paracrine manner from mesenchymal cell types to epithelial cells. Successful ligand binding results in phosphorylation of the intracellular tyrosine domains on FGFR, which in turn activates downstream signaling including Ras-MAPK and PI3K-Akt pathways, resulting in mitogenic and proliferative responses within the cell.

During ductal elongation, FGF1, FGF2, FGF4, FGF7 and FGF10 are all highly expressed in the mammary gland [94]. Overexpression of Fgf4 under control of the WAP promoter inhibits ductal elongation and side branching in conjunction with causing structural defects in the TEB including large lumen, a lack of a neck region and cap cell layer, and layers of cells filling the lumenal space. These TEBs also exhibit decreased level of apoptosis [96]. Fgf7 also seems to play an inhibitory role in branching possibly through opposition to TGFalpha signaling [97].

The best characterized FGF ligand-receptor pair is FGF10/FGFR2b. Fgf10 is produced by the adipose tissue and acts in a paracrine manner on the mammary epithelium. Fgf10 ligand is localized in the mesenchyme near ducts and alveoli, while its receptor Fgfr2b is localized to the TEB (Fig. 5c) [98]. Fgf10 binds Fgfr2b in a specific manner with heparan sulfate as a required cofactor [99]. Conditional knock out (KO) of FGFR2b in the mammary epithelium immediately following birth and continuing into the first few weeks of puberty results in failure to form TEBs or to grow past the lymph node [95]. This is due to a large decrease in proliferation in the luminal epithelium specifically and an increase in apoptosis. This seems to be only a temporary block, as withdrawal of doxycycline results in formation of TEBs and successful outgrowth, indicating that FGF10 signaling is not required to maintain the regenerative ability of the mammary epithelium [100]. Further studies have shown that Fgfr2 is required in the basal compartment for regeneration of a gland after transplantation, and that basal cells lacking Fgfr2 fail to give rise to luminal progeny [95]. Interestingly, in vitro work has indicated that Fgf10 from a point source acts as a chemoattractant for MECs and may provide a directional cue for TEB growth in vivo [53].

### Transforming Growth Factor Beta

Transforming Growth Factor Beta (TGFβ1, TGFβ2, TGFβ3) ligands are secreted as a latent complex that is activated by cleavage by proteases including MMP-9, thrombospondin, or plasmin [101]. Therefore they are often found in the extracellular matrix, which acts as a reservoir. When activated, the ligand binds its receptor, TGFβRII and TGFβRI, which results in internal kinase activity and the activation of SMAD dependent and independent signaling cascades. Eventually, this activation results in various downstream actions including gene transcription, motility, invasion, apoptosis, proliferation, and ECM remodeling. The final physiological response to TGFβ signaling depends on the expression of other transcription factors and signaling proteins that work in conjunction with SMAD proteins.

In the mammary gland, TGFβ isoforms are expressed in the epithelium at all phases of development and generally act as inhibitors of proliferation and promoters of ECM deposition [102]. TGFβ1 and TGFβ3 have overlapping expression patterns in the epithelium during ductal elongation, although TGFβ3 is the only isoform found within the cap cell layer of the TEB. TGFβ2 is expressed at very low levels in the TEB and is upregulated during pregnancy. Initially, TGFβ ligands were shown to inhibit the growth of TEBs when ectopically expressed [103, 104]; however, it has since been shown that the levels of TGFβ have divergent affects. Low levels (femtomolar) of TGFβ stimulate branching morphogenesis , and higher levels (picomolar) inhibit growth [105]. Interestingly, TGFβ signaling in stromal cells induces proliferation and production of ECM (Fig. 5d) to contribute to TEB regression [106]. High estrogen levels during puberty inhibit TGFβ ligand expression, resulting in low TGFβ levels during ductal elongation. Overexpression of an activated form of TGFβ1 during ductal elongation results in hypoplastic growth [107], whereas glands from TGFβ1 heterozygotes have a two-fold increase in ductal elongation and proliferation within TEBs [108]. Additionally, deletion of TGFβ1 in mouse epithelium resulted in aneuploidy, centrosome aberrations, and impaired DNA damage response (DDR) through crosstalk between SMADs and ataxia telangiectasia mutated (ATM), a protein kinase that is activated by DNA double-strand breaks. This aspect of TGFβ function is especially interesting in a breast cancer context, in which TGFβ-resistant cells may be under positive selection. This role of TGFβ has not been explored in the TEB, although maintenance of DDR would be especially important in such a highly proliferative structure [109].

### WNT

There are 19 identified Wnt ligands and 10 Frizzled receptors as well as several co-receptors [110], which have been the subject of intensive research in the mammary gland for over two decades ([111] and references therein). The Wnt pathway can be activated by both canonical and non-canonical ligands. In the canonical pathway, Wnt ligands bind to a Frizzled receptor and a low-density lipoprotein receptor-related protein 5 or 6 (LRP). This association causes the disassembly of a multiprotein complex containing glycogen synthase kinase 3 (GSK3), casein kinase 1α (CK1), axin and adenomatous polyposis coli (APC), and β-catenin; allowing the β-catenin to translocate into the nucleus and bind LEF transcription factors. The non-canonical pathway does not involve LRP coreceptors or the stabilization of β-catenin, but rather can result in changes in cell polarity and cytoskeletal rearrangements through the planar cell polarity pathway (PCP) or result in the release of intracellular calcium through the calcium pathway. The PCP pathway results in downstream activation of disheveled, JNK and Rho family GTPases, which direct asymmetrical cytoskeletal rearrangements and cellular polarity. In the calcium pathway, Frizzled acts through G-proteins to activate phospholipase C (PLC) which results in release of intracellular calcium and eventual activation of the transcription factor NFAT [112]. Additionally, the non-canonical pathways can signal through alternate Ror and Ryk receptors.

Wnt pathway components are temporally and spatially differentially expressed in the mammary gland [113, 114]. For instance, the LRP6 co-receptor is expressed in both layers of the epithelium and stroma during embryogenesis; however, in the juvenile and adult, expression in the epithelial compartment localizes to the basal layer only [115]. Further, ligands Wnt4 and Wnt5a are localized to the luminal compartment, whereas Wnt6 is localized to the basal layer. The cognate non-canonical receptors for these ligands follow similar patterns of expression [50].

Canonical Wnt signaling is required for stem cell maintenance, branching, and alveologenesis in the mammary gland. Canonical signaling has been shown to be important in TEBs because Lrg5 and 6 are required for canonical signaling and are localized to the basal layer (Fig. 5e). With targeted deletion in mammary glands, fewer TEBs develop and glands have diminished side branching [115, 116]. The LRP5 co-receptor is also important in the luminal layer for stem cell function and regenerative ability during transplantation [117]. Similarly, Wnt4, has been shown to act through the canonical pathway specifically in the myoepithelial cells starting in the neck of the TEB. Canonical signaling was also seen in the TEB and the stroma directly surrounding the TEB but was not Wnt4 dependent [118].

Non-canonical Wnt signaling also plays an important role in the developing mammary gland. In situ hybridization has shown the specific expression of Wnt2, Wnt5a, and Wnt7b in the TEB during puberty. Wnt5a, a non-canonical Wnt ligand, regulates branching and proliferation within the TEB by inhibiting Wnt/β-Catenin activity [119]. Interestingly, the non-canonical receptor Ror2 has been shown to be important for normal TEB morphology and for controlling proliferation. Ror2 expression shows an inverse relationship to Wnt activity in the TEB, where Ror2 is expressed in the neck and subtending duct and β-catenin activity occurs in the cap cell layer of the TEB. Knockout of Ror2 results in increased branching during outgrowth, decreased percent fat pad filled, and budding in the neck of the dysmorphic TEBs. Roarty et al., concluded that Wnt/β-catenin activity and Ror2 expression oppose each other in the TEB to maintain stemness in progenitor populations in the cap cell region, inhibit proliferation in committed cells in the neck of the TEB, and promote differentiation of the myoepithelial cell type throughout the TEB (Fig. 5e).

Other Wnt ligands and receptors remain unstudied in mammary gland development, and further detailed studies are needed to fully understand this complex signaling axis in the TEB.

### Axon Guidance Molecules

There are many other signaling molecules being explored as potentially important for mammary gland development. These include SLIT/ROBO and Netrin1/Neogenin. These molecules are members of the axon guidance genes most well known for their role in axonal growth and migration. More recently it has become clear that they play important roles in tissue morphogenesis in diverse organ types including the mammary gland.

SLIT2 is expressed in both the body cell and cap cell layer of the TEB; however, its receptor, ROBO1, is expressed exclusively in the cap cell layer (Fig. 5f). In both SLIT2 and ROBO KO studies TEBs show severe abnormalities, including separation of the cap and body cell layers, invagination of the cap cell layer, and disorganization of the body cell layer. Interestingly, Netrin1/Neogenin KO mice have nearly identical phenotypes [120]. Netrin1 KO mice do not exhibit cap cell invaginations, although they do exhibit an increase in the number of cap cells present in the body cell layer, and an increase in overall cap cell death. Further studies of the Slit2/ROBO1 pathway have determined that Slit2 regulates the expression of Inscuteable (mInsc), which is responsible for regulation of asymmetric versus symmetric cell division in the cap cell layer. Ballard et al. showed that increased levels of mInsc causes increased outgrowth during puberty due to an expansion of the stem cell compartment presumably via an increase in symmetrical divisions, however these mice did not exhibit any morphological changes in the resulting outgrowths as one would expect from an expansion of the stem cell compartment [121].

Other genes involved in axonal guidance are known to be expressed or enriched in the TEB, including brain acid-soluble protein 1 (BASP), small proline rich protein 1A (sprr1A), and semaphorin 3B [122]. However, they have not been functionally or specifically studied during puberty. Further work in this area promises to uncover interesting details in TEB biology.

## Mechanisms of Forward Growth

The exact mechanism of TEB forward movement has not been completely elucidated. One theory is that mechanical pressure from ECM production and stromal condensation in the neck of the TEB in conjunction with high proliferation rates within the body cell layer combine to cause net forward movement. In vitro studies have recently indicated that a collective migration mechanism may also be important for TEB forward progress, and of course matrix degrading proteins secreted by the TEB itself are thought to be important for creating a path of least resistance. Together, these characteristics make the TEB a unique structure in organogenesis, which may provide valuable insight into the understanding of other migratory and invasive groups of cells including cancers.

1. Collective Migration

Collective migration is the process that enables the coordinated movement of groups of cells that retain their cell-cell junctions. Collective migration requires the coordination of adhesion, polarization, and mechanocoupling to sense external guidance cues that can then be transmitted throughout the group of cells. It also involves the establishment of leader-follower relationships, in addition to the chemical guidance mechanisms seen in individual cell movement [123].

Studies by Ewald et al. have demonstrated a novel mechanism by which mammary ducts elongate. While the TEB has hallmarks of collective migration, a major distinction in the TEB is the lack of subcellular protrusions at the leading edge. TEBs instead have a completely smooth surface at the leading edge, which makes this structure unique. Interestingly, cells in the interior of the body cell layer exhibit characteristics of active migration with both protrusions and active motility observable by live imaging [28].

Ewald et al. also used an organoid model to study elongation and branching in vitro. They found that mammary organoids go through distinct stages of elongation. First, organoids form polarized bilayers with large clear lumens. Second, they undergo luminal filling to form a complex cyst similar in morphology to a TEB in vivo. Finally, organoids undergo proliferation-dependent elongation. Elongating regions are always multilayered with thinning and reversion to a simple bilayer at sites adjacent to the elongating region---resembling the bulb morphology of a TEB. Upon closer inspection, Ewald et al. observed the chaotic movement of both luminal and myoepithelial cells in the leading tip during elongation [27] [28]. The luminal cells constantly moved in a forward direction, but myoepithelial cells moved in both forward and reverse directions.

In addition to the behaviors above, Ewald et al. also observed bifurcation events that occurred only at areas with partial coverage by myoepithelial cells, whereas complete coverage resulted in cessation of elongation [27]. Interestingly, unlike previous descriptions of collective migration, the cells at the advancing tip never exhibited cellular extensions or actin-rich protrusions. Instead, the cells continuously exchanged positions while maintaining a completely smooth edge [27]. Further studies using transmission electron microscopy (TEM) confirmed the lack of even fine protrusions from the basal layer into the ECM, as well as from the luminal layer into the ECM. There were, however, complex interdigitations between body cells in the interior of the organoids and within TEBs in vivo. These extensive cell membrane structures are both simple and branched in different areas and contain desmosome attachments between neighboring cells [27, 28].

It is interesting to note that the complex interdigitation seen in the body cell layer of the TEB is similar to the morphology of atypical ductal hyperplasias, intraductal carcinomas, and infiltrating ductal carcinomas [28, 124], providing an additional link between characteristics of the TEB and those of invasive breast tumors. The TEB may therefore be a useful model for the specific study of these interdigitations, and their role in cell communication, invasion, and mobility, particularly as these functions relate to breast cancer.

2) Epithelial to Mesenchymal Transition

Epithelial to mesenchymal transition (EMT) is a normal process involved in embryogenesis and organogenesis in which polarized epithelial cells undergo biochemical changes to assume a mesenchymal cell phenotype. There are three classifications of EMT; type 1 includes normal changes during implantation and organogenesis, and does not result in invasive phenotypes; type 2 is associated with wound healing and regeneration and results in fibrosis and inflammation; and type 3, which is reserved for neoplastic cells and results in invasion and metastasis of the primary tumor. Many signaling pathways involved in the regulation of EMT regulation are also present in the TEB including Wnt, FGF,TGFβ [125], Slug and Snail [126].

Motile cells within the TEB seem to have undergone a partial EMT due to reduced polarity, reduced adhesion, and increased motility characteristics. Indeed, the cells within the body cell layer only contain desmosomal adhesions, but interestingly, these seem to play an inhibitory role in ductal elongation since chemical inhibition of desmosomal proteins causes increased branching [127]. Additionally, cells within the TEB express markers of EMT including Slug, Zeb1, and Twist [128]. The increased mobility endowed by the EMT in body cells may provide additional support for the forward movement and invasion of the fat pad during ductal elongation.

3) Matrix Metalloproteinases and Heparanase Production

The TEB expresses several proteins that aid in forward movement. The cap cell layer expresses MMPs and heparanase [129], which selectively degrade the ECM immediately in front of the TEB in order to create a path of least resistance, possibly to aid in forward movement. It has recently been shown that MMP-14 and heparanase form a reciprocal positive feedback loop in the TEB that increases branching. Gomes et al., showed that knock down of heparanase in vivo and in vitro results in decreased branching during ductal outgrowth concomitant with a decrease in MMP-14 levels, and vice versa [129]. Further, work by the same group has shown through selective domain deletion studies that MMP-14 has a non-proteolytic role in branching morphogenesis through binding to integrin β1 and activating the MAPK signaling pathway. They also presented evidence that MMP-14, integrin β1, and MAPK pathway activation form a three-way regulatory loop. Together, these data indicate a complex interaction between MMP-14, integrin, heparanase, and the MAPK pathway activation that is essential for successful TEB invasion and ductal elongation [130].

A downstream target of MMP-14, MMP-2, is also involved in ductal outgrowth during puberty. Although TEBs have a normal morphology, size, proliferation rate, and basement membrane composition, MMP-2 KO mice have a greatly increased number of apoptotic cells, which is thought to inhibit the ability of these TEBs to invade the fat pad by mitigating the forward pressure from proliferation [131]. MMP-11 KO mice exhibit a similar phenotype to MMP-2 KO mice in that they exhibit defective ductal outgrowth, with ducts never filling the fat pad, whereas the morphology of the TEBs is normal. Together these data indicate a role for MMPs in the ability of TEBs to invade the fat pad, but not necessary for proper TEB morphology [130].

4) Basement Membrane Deposition

The cap and myoepithelial cells deposit the basement membrane during ductal elongation, which results in both polarization of the luminal layer and geometric confinement of the subtending duct. Laminin-1 expressed by the myoepithelial cells [8, 29] has been shown to be required for proper polarization of luminal cells in culture, the absence of which contributes to disorganization in breast cancers [132]. The tip of the TEB is covered in hyaluronic acid and laminin; however, starting in the neck of the TEB, the basement membrane becomes a thick meshwork of collagen type IV, laminin 1 and 5, and heparin sulfate proteoglycans [30]. Fibroblasts, adipocytes, endothelial cells and immune cells also contribute to the production of ECM [133]. Not only the composition of the ECM but also its orientation seems to be important for ductal morphogenesis as collagen fibers radiate out from the TEB and ductal development follows topographical cues in the ECM [54, 134, 135].

In vitro studies have also indicated that mammary acini rotate constantly in space as laminin is deposited, and that this rotation is dependent on both correct polarization of the cells and deposition of laminin-1 and collagen IV [136]. Whether this rotation aids in forward movement during ductal elongation has not been verified, but it is possible that TEBs move in a corkscrew type manner in order to better invade the stroma.

## Terminal End Buds as Models for Cancer

**Fig. 6:**
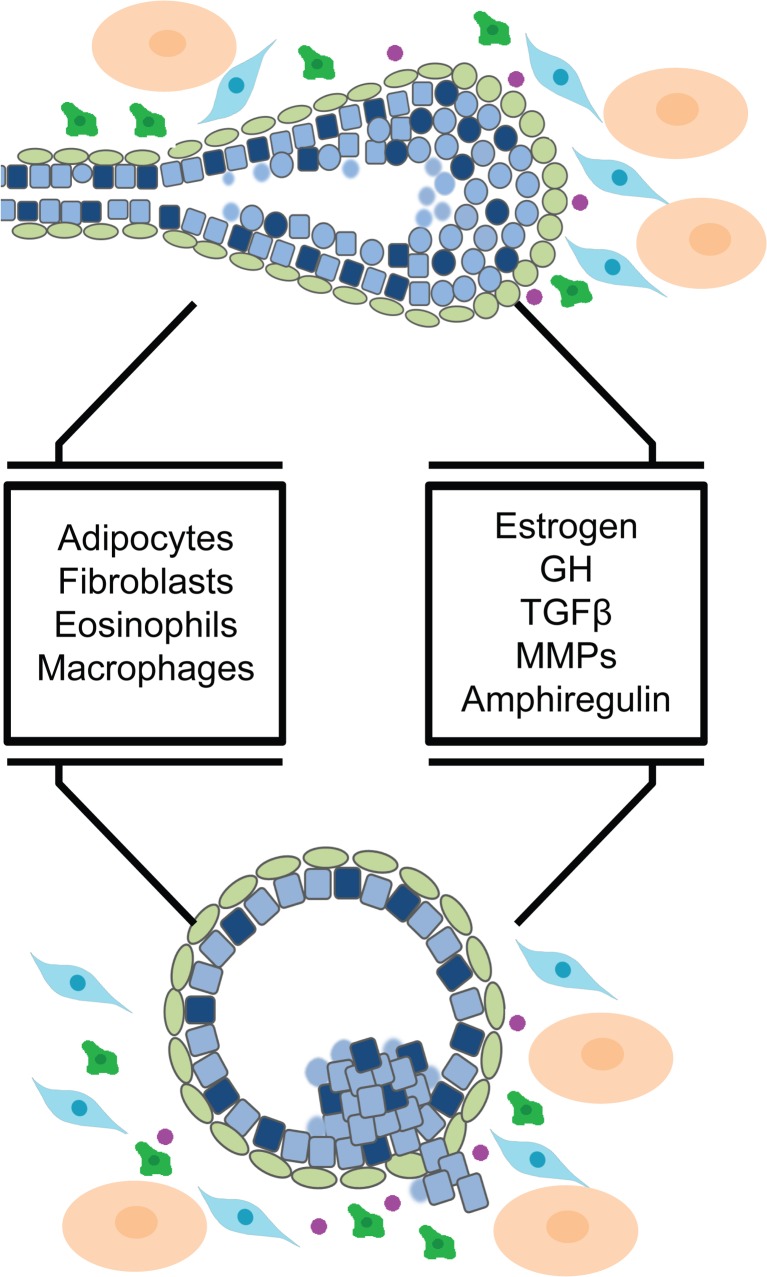
The Terminal End Bud as A Model for Breast Cancer. The terminal end bud has many important features in common with early breast cancers including close contact with specialized stromal cell types, and several signaling pathways that are important for cell growth and survival

Although the TEB is a normal structure and behaves in an organized manner, many of the biological processes involved in its growth are also required for the abnormal growth of tumors. As such, the TEB can be used as model for the study of several aspects of breast cancer biology (Fig. 6). First, like breast cancers, TEBs have extremely high proliferation and apoptosis rates and a heterogeneous cell population [34, 49, 137], comparable to breast tumors [138–140]. Second, the TEB recruits its own blood supply as it grows through the nascent fat pad, causing blood vessels to form around the ducts [59]. This is most likely through the recruitment of macrophages, which in turn, recruit endothelial cells, but also through the epithelial expression of vascular endothelial growth factor (VEGF) [141]. Tumors are known to also recruit blood vessels that aid in their continued growth through the overexpression of VEGF [142, 143]. Third, the TEB is an invasive structure, with a unique basement membrane composition and thickness, which allows for specialized interaction with the mammary stroma. During tumor invasion, factors that are expressed normally in the TEB, such as MMPs, are dysregulated to degrade the basement membrane and facilitate invasion and interaction with the stromal cell types directly [144]. Finally, a relatively unstudied commonality between TEBs and abnormal growths are micro-scale cellular inter-digitations unique to body cells and hyperplasias [27, 124], the functions of which remain unclear.

The TEB is also more highly susceptible to early oncogenic insult resulting in tumors later in life. Studies in rats indicated that glands exposed to 7,12-dimethylbenz(α)anthracene (DMBA) during puberty formed tumors at a higher rate than rats exposed at older ages [14]. This is most likely due to the high proliferation rate and accelerated cell cycle unique to the TEB, in both rats and mice, which may compromise the ability of repair machinery to work in these cells [15, 40]. It remains unclear whether homologous recombination (HR) or non-homologous end-joining (NHEJ) is the favored repair mechanism in the TEB although MaSCs have been shown to have increased, error-prone, NHEJ activity [145].

One caveat to the use of the TEB as a model of cancer are the apparent differences between the collective migration invasion mechanism of the TEB and the single cell active migration exhibited by cells within DCIS as it transitions into invasive breast cancer. However, with respect to what aspect of tumor/TEB biology to study, whether the investigator is interested in molecular signaling (MMPs, adherens junctions, VEGF, etc.), epithelial-stromal interactions (fibroblasts, macrophages), ECM composition and integrity, or gene expression, the TEB is an under-utilized model system.

## Conclusion

The TEB remains a powerful “experimental organism” for the study of developmental phenomena and may provide valuable insight into cancer dynamics. Data indicate that the TEB represents a unique example of 3D collective migration. Not only do the internal cells within the TEB exhibit active migration, but the combination of adhesion molecule expression (P- and N-cadherin), secretion of ECM remodeling factors (heparanase and MMP-14), and basement membrane deposition provide key effects that together lead to fat pad invasion. Further, the TEB represents a unique environment for molecular signaling studies, as it responds to systemic factors, paracrine signals, and physical signals from the ECM.

Several aspects of TEB biology remain undefined. One aspect that still remains unclear is the role of topographical instructive cues such as collagen tracks, which may help to direct ductal elongation. Additionally, the regulation of the transition from the bulbous form of the TEB into a simple bilayer of the mature duct is not fully understood. While there has been an effort to tease out the molecular signaling pathways involved in TEB formation, maintenance, and regression, there is still much work remaining in order to understand the complex dynamics of this structure.

## Abbreviations

TEB: terminal end bud
TDU: terminal ductal unit
ECM: extra cellular matrix
sSHIP: SH2-containing inositol 5’-phosphatase
FGF: fibroblast growth factor
IGF-1: insulin-like growth factor
HGF: hepatocyte growth factor
MMPs: metalloproteinases
TIMPs: tissue inhibitors of metalloproteinases
CSF1: colony stimulating factor 1
EGF: epidermal growth factor
Areg: mphiregulin
VEGF: vascular endothelial growth factor
GH: growth hormone
TGFb: transforming growth factor beta
ESR1: estrogen receptor alpha
P4: preogesterone
PR: progesterone receptor
RANKL: nuclear factor kappa B ligand
EGFR: epidermal growth factor receptor
MAPK: mitogen activated protein kinase
PI3K: phosphoinositide 3-kinase
WAP: whey acidic protein
Fgfr2b: fibsoblast growth factor receptor 2 b
DDR: DNA damage response
ATM: ataxia telangiesctasia mutated
LRP: lipoprotein receptor-related protein
GSK3: glycogen synthase kinase 3
CK1: casein kinase 1alpha
APC: axin and adenomatous polyposis coli
PCP: planar cell polarity
JNK: c-Jun N-terminal kinase
PLC: phospholipase C
NFAT: muclear factor of activated T-cells
ROBO: roundabout
mInsc: inscuteable
BASP: brain acid-soluble protein 1
Sprr1A: small proline rich protein 1A
TEM: transmission electron microscopy
EMT: epithelial to mesenchymal transition
DMBA: 7,12-dimethylbenz(alpha)anthracene
HR: homologous recombination
NHEJ: non-homologous end-joining
MaSC: mammary stem cell
DCIS: ductal carcinoma in situ
